# Case Report: A diabetic ketoacidosis in adolescents complicated with rhino-orbital-cerebral mucormycosis

**DOI:** 10.3389/fmed.2026.1847375

**Published:** 2026-07-14

**Authors:** Jinxin Tang, Qishu Hou, Jifeng Ye, Yueyue Weng

**Affiliations:** 1 Department of Pharmacy, Wenzhou Central Hospital, Wenzhou, China; 2 Department of Pharmacy, The Second Affiliated Hospital and Yuying Children's Hospital of Wenzhou Medical University, Wenzhou, China

**Keywords:** adolescent, amphotericin B, isavuconazole, mucormycosis, rhino-orbital-cerebral mucormycosis

## Abstract

**Background:**

Rhino-orbital-cerebral mucormycosis (ROCM) is an aggressive infection caused by Mucorales fungi, with rapid progression, high mortality, and disability. The insidious onset and atypical early symptoms predispose the condition to misdiagnosis or delayed diagnosis. There are no evidence-based guidelines for the diagnosis and treatment of mucormycosis in children. This case report describes the entire diagnostic and treatment process for a 14-year-old female patient with type 1 diabetes mellitus (T1DM) who developed ROCM following diabetic ketoacidosis (DKA). It highlights the key role of clinical pharmacists in multidisciplinary collaboration, pharmacological evaluation, antifungal regimen optimization, and medication monitoring.

**Case description:**

A 14-year-old female patient was admitted to the hospital due to persistent chest pain for 5 days and was diagnosed with T1DM complicated with DKA. During the treatment of DKA, the patient suddenly developed high fever, nasal congestion, progressive eyelid and periorbital swelling, headache, and cranial nerve dysfunction. Plasma mNGS and nasal mucosa biopsy both detected Rhizopus delemar, confirming ROCM. The initial treatment was liposomal amphotericin B (5 mg/kg/d) combined with nasal endoscopic debridement, the infection was initially controlled. However, on the 16th day, anemia aggravated (Hb 84 g/L), and severe hypokalemia (K^+^ 2.41 mmol/L) occurred. After a multidisciplinary assessment led by a clinical pharmacist and completion of a 4-week course of liposomal amphotericin B, the treatment plan was adjusted to include oral isavuconazole and intranasal amphotericin B deoxycholate. Follow-up for 4 months, facial and periorbital swelling completely resolved, olfaction recovered, facial sensation partially recovered, and no significant adverse drug reactions occurred.

**Conclusion:**

This case demonstrates that in adolescent patients with T1DM who develop ROCM following DKA, non-specific prodromal symptoms such as headache, facial swelling, and nasal congestion should be closely monitored. Early implementation of a multi-modal diagnostic approach combining mNGS and tissue biopsy is necessary. Based on organ function, drug toxicity, and pharmacokinetic characteristics, individualized antifungal regimens that balance efficacy and target organ protection should be formulated. Clinical pharmacists play a crucial role in the management of severe fungal infections by participating in diagnostic collaboration, regimen optimization, and continuous safety monitoring, thereby enhancing the scientificity, safety, and precision of treatment.

## Introduction

Mucormycosis is a rare and invasive infection caused by fungi in the order Mucorales, with an annual global incidence rate of approximately 2.7 per 100,000 and a mortality rate of 39.8% (1). It is classified into various types based on the site of infection, including rhino-orbital-cerebral mucormycosis (ROCM), pulmonary, cutaneous, renal, gastrointestinal, and disseminated types (2). ROCM is the most common type (accounting for 50%–70%), and it is prone to vascular invasion and central nervous system involvement, resulting in the highest rates of disability and mortality, making it the most destructive subtype (3). Mucormycosis in children is extremely rare, with the literature largely comprising case reports and a paucity of high-quality evidence. In clinical practice, adult guidelines are often applied, leading to delayed diagnosis, non-standard treatment, and insufficient monitoring of drug safety. This case report describes the entire diagnosis and treatment process of a 14-year-old T1DM patient with ROCM secondary to DKA, highlighting the crucial role of clinical pharmacists in individualizing antifungal regimens, early identification and intervention of adverse reactions, and structured pharmaceutical care from hospitalization to follow-up, providing practical references for multidisciplinary management and clinical pharmacy services in severe fungal infections in children.

## Case information

A 14-year-old female patient (weight 38.5 kg, height 153 cm) was admitted to our hospital on September 22, 2025, due to “repeated chest pain for 5 days.” 5 days ago, she suddenly experienced chest pain behind the sternum without any obvious cause, each episode lasting from several minutes to half an hour, which could be partially relieved by rest. She also experienced chest tightness and occasional labored breathing, but no cough, hemoptysis, syncope, or consciousness disorder. A chest CT at an outside hospital showed multiple gas shadows in the neck and mediastinum, and she was diagnosed with “cervical and mediastinal emphysema.” Past medical history: Hashimoto’s thyroiditis for 2 years, long-term oral administration of levothyroxine sodium tablets (25 μg/d), denies any other medical history.

Admission physical examination: T 36.8 °C, P 122 beats/min, R 24 beats/min, BP 133/82 mmHg; Conscious, but listless; No pallor, jaundice, or petechiae on the skin and mucous membranes. The lungs have slightly coarse breath sounds, no rales or wheezes. No subcutaneous emphysema was palpable in the anterior chest and neck, nor was there any tenderness. Auscultation of the heart and lungs, abdominal examination, and neurological examination all revealed no significant abnormalities.

On the first day of hospitalization, a chest CT scan showed a small amount of emphysema in the anterior mediastinum, which had decreased compared to before. Blood gas analysis indicated metabolic acidosis (pH 7.08, PaCO2 15 mmHg, BE −23.7 mmol/L, lactate 1.45 mmol/L). Diabetes-related antibodies were positive: anti-glutamic acid decarboxylase antibody (anti-GAD Ab) 408.08 IU/mL, Anti-tyrosine phosphatase antibody (IA-2A) 5.81 IU/mL, anti-insulin antibody (IAA) 0.07 COI, anti-islet cell antibody (ICA) 4.77 COI. Glycated hemoglobin A1c was 12.54%, C-peptide was 0.19 ng/mL, blood glucose was greater than 30 mmol/L, and urine routine showed glucose (4+), ketone bodies (4+), and occult blood (2+). Serum potassium was 2.83 mmol/L, CRP was 11.55 mg/L, WBC was 13.95 × 10^9^/L, Hb was 112 g/L, and PLT was 241 × 10^9^/L. Dynamic biochemical profiles during hospitalization and follow-up are listed in Table 1. Upon further inquiry into the medical history, the child had experienced polydipsia and polyuria for nearly 20 days and had lost 4 kg in weight in the past half month. Supplementary diagnosis: T1DM, DKA. Treatment included fluid expansion with normal saline, insulin infusion (0.1 U/kg/h), potassium supplementation, and cefuroxime for anti-infection.

**Table 1:** Dynamic biochemical profiles during hospitalization and follow-up.

The laboratory parameters	Unit	DAY1	DAY6	DAY8	DAY10	DAY13	DAY16	DAY20	DAY23	DAY29	DAY30	DAY36	DAY42	DAY66	DAY87	DAY136
CRP	mg/L	11.55	42.21	103.56	129.43	54.04	20.22	12.84	16.72	11.34	11.83	5.76	3.8	NP	NP	NP
Hb	g/L	112	101	90	85	90	84	76	82	83	86	94	105	101	102	100
Na^+^	mmol/L	133	NP	131.4	135.6	136.3	140.5	142.3	141	NP	142	NP	138	139	140	140
K^+^	mmol/L	2.83	NP	4.48	3.31	3.3	2.41	2.59	2.84	3.29	3.38	4.11	4.32	4.29	4.42	4.54
ALT	U/L	13	NP	NP	12	12	NP	NP	15	NP	16	16	13	8	12	20
Cr	μmol/L	51	NP	NP	40.4	58	NP	NP	49	NP	47	60	69	63	57	59

On the 6th day of hospitalization, the child developed a fever (peak temperature 38.2 °C), CRP rose to 42.21 mg/L, WBC was 16.96 × 10^9^/L, Hb was 101 g/L, G test was 153.17 pg./mL, GM test was less than 0.1 ng/mL, and sputum culture showed *Klebsiella pneumoniae* (sensitive to ceftriaxone). The anti-infection treatment was adjusted to ceftriaxone (2 g qd ivgtt). The child’s blood glucose fluctuated between 5.0–13.7 mmol/L, and the hypoglycemic regimen was adjusted to 5 U of insulin aspart subcutaneously before each meal and 12-14 U of insulin glargine subcutaneously before bedtime.

On the 8th day of hospitalization, the child still had a fever (peak temperature 38.6 °C), accompanied by nasal congestion, epistaxis, and numbness around the mouth. The CRP level rose to 103.56 mg/L. The antibacterial drug was upgraded to meropenem (1 g q8h ivgtt).

**Figure 1:**
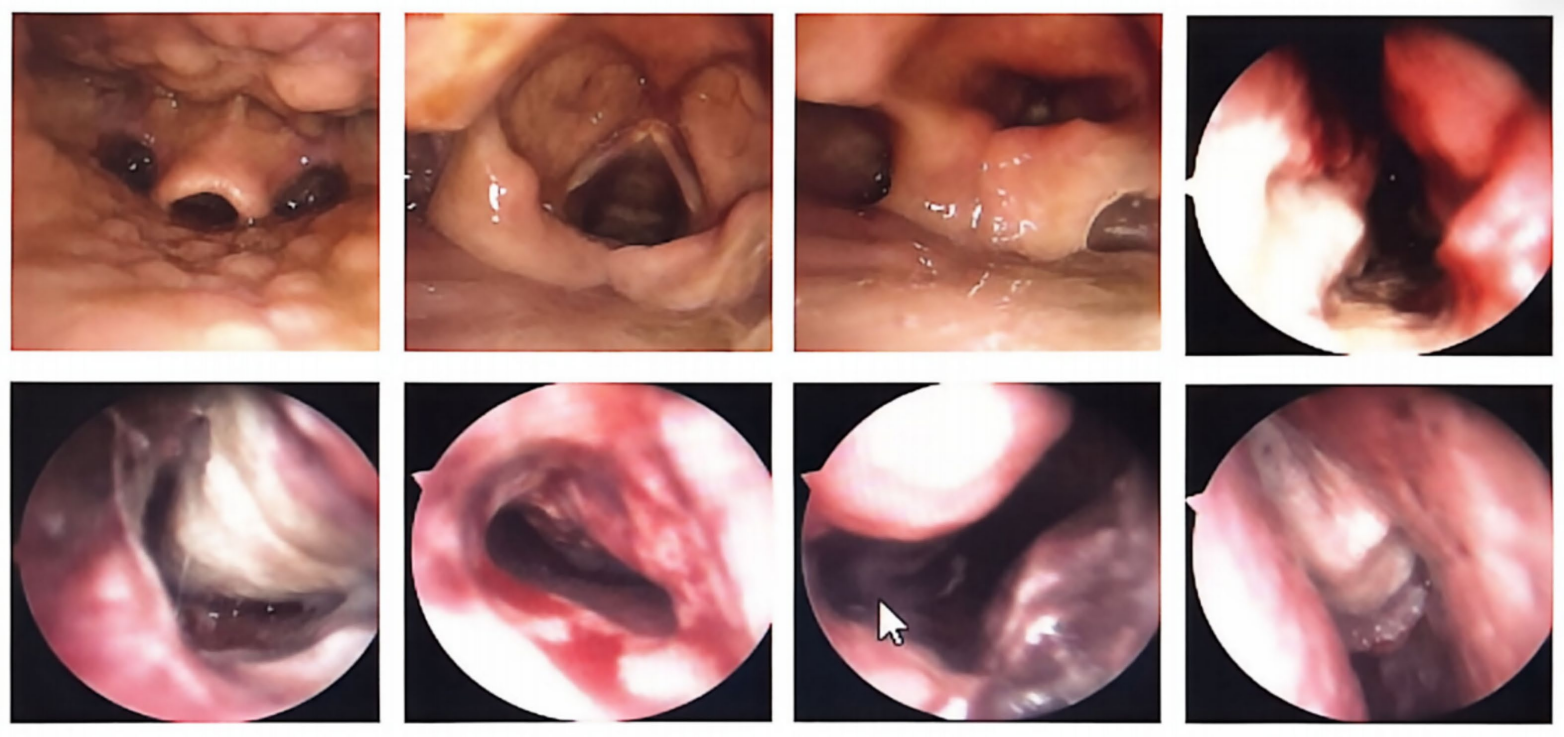
Nasal endoscopy on hospital day 9.

On the 9th day of hospitalization, the child continued to have a high fever (peak temperature 39.2 °C), with progressive swelling of the eyelid and periorbital area, and aggravated nasal congestion and epistaxis; physical examination showed decreased touch and smell sensation from the ala nasi to the upper lips; nasal endoscopy revealed grayish-white pseudomembranous necrotic material. After 48 h of meropenem treatment, CRP rose to 122.65 mg/L; G tese was 78.72 pg/mL, GM test was 0.12 ng/mL. Considering the clinical manifestations, imaging background, and microbiological clues, invasive fungal infection was highly suspected, and mucormycosis could not be excluded. On the same day, empirical antifungal treatment was initiated: liposomal amphotericin B (200 mg qd ivgtt), and nasal mucosa biopsy (pathology) and peripheral blood mNGS testing were performed (Figure 1).

**Figure 2:**
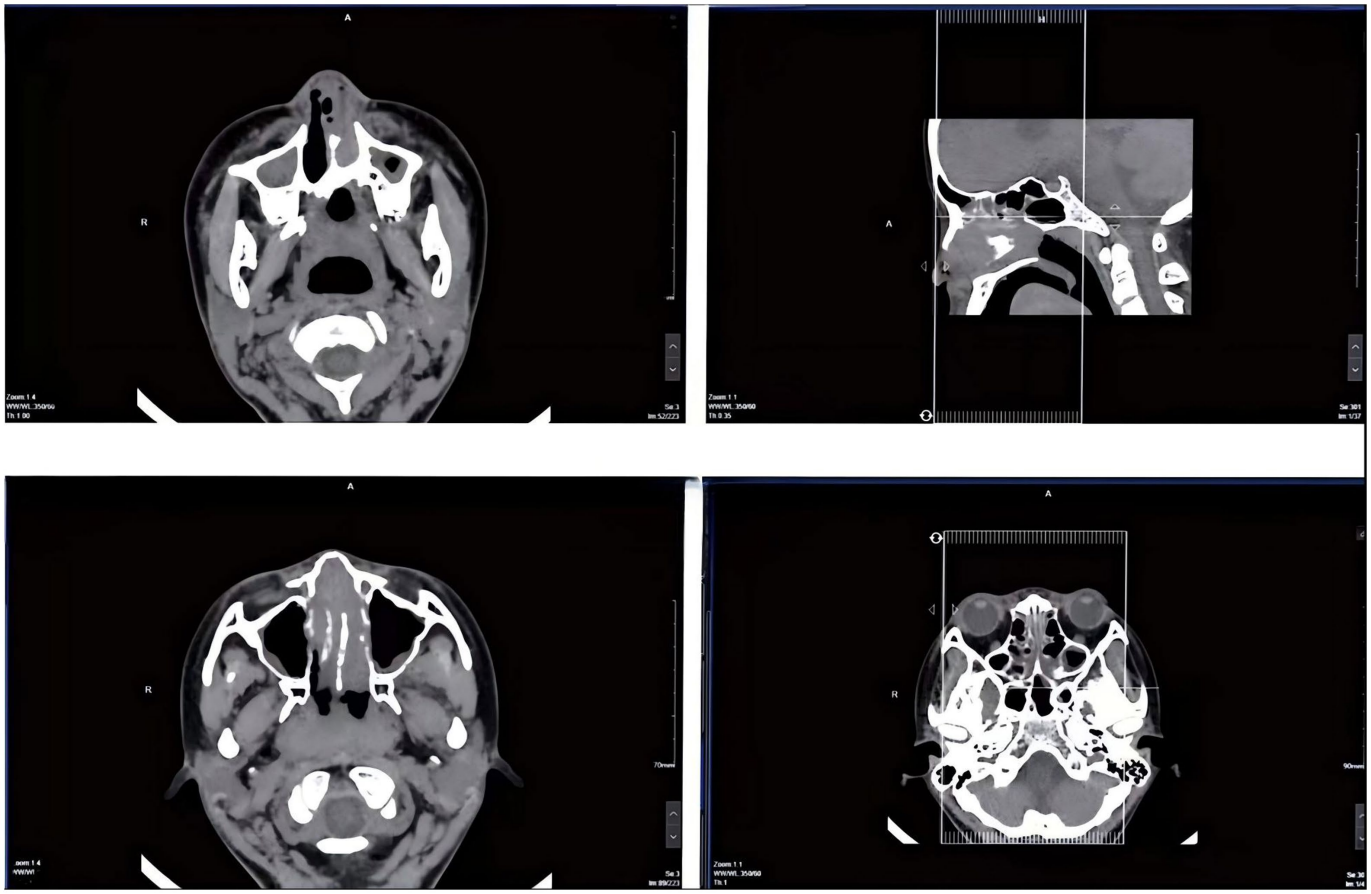
Sinus CT on hospital day 11.

**Figure 3:**
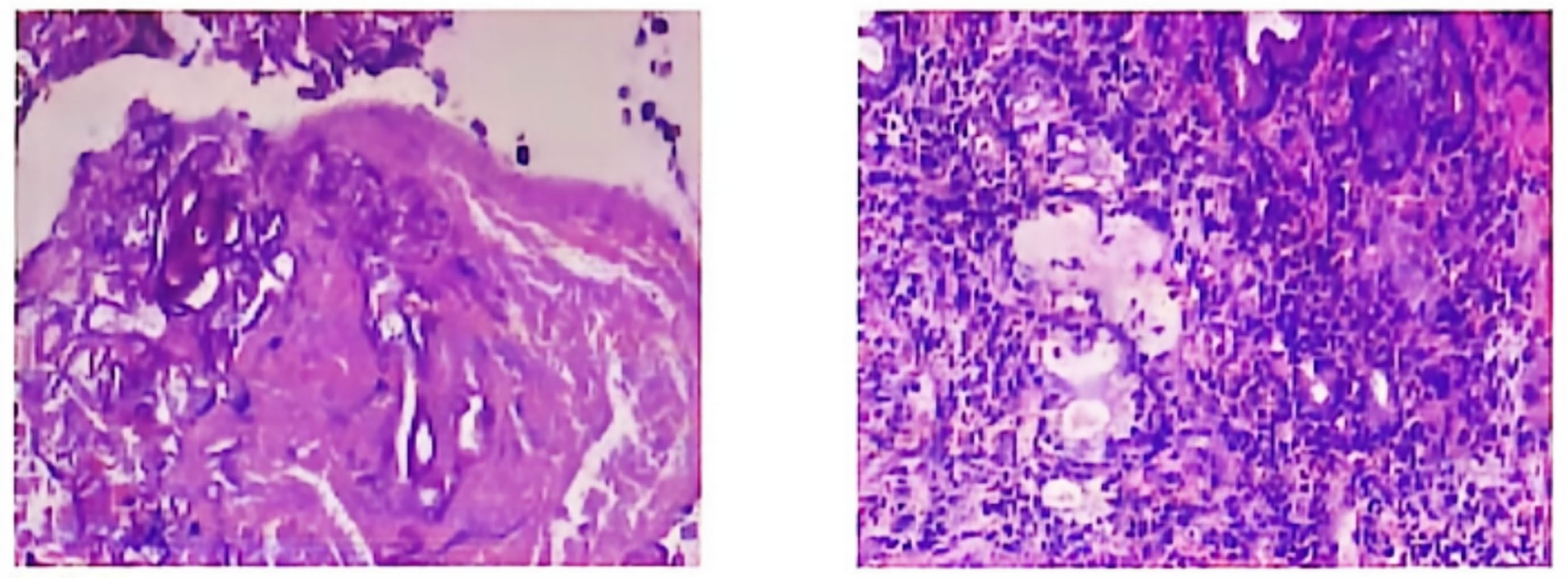
Nasal mucosal pathology findings.

On the 12th day of hospitalization, the child’s body temperature returned to normal. Still, the child showed apathy, listlessness, swelling in the facial triangle, and loss of sensation and smell from the ala nasi to the upper lip. mNGS of peripheral blood detected Rhizopus delemar (sequence count 639), and the pathological examination of nasal mucosa tissue showed fungal hyphae in the necrotic tissue, which was consistent with mucormycosis. Sinus CT showed no obvious bony destruction (Figure 2). Although no abnormalities were found on cranial MRI, combined with sensory disturbance of the maxillary nerve, apathy, mild electroencephalographic abnormalities, and a slow background rhythm, early intracranial invasion was considered, and the diagnosis of ROCM was clear. On the same day, the child underwent “endoscopic resection of nasal cavity and paranasal sinus lesions + resection of nasal septum lesions + septal cartilage bone harvesting surgery + partial resection of bilateral inferior turbinates” under general anesthesia to thoroughly remove the necrotic tissue. The postoperative pathological report showed: acute and chronic inflammation of small pieces of tissue mucosa, fungal hyphae in the necrotic tissue, consistent with mucormycosis; special staining showed acid-fast (−), Grocott methenamine silver (+), and periodic acid-Schiff staining (+) (Figure 3).

**Figure 4:**
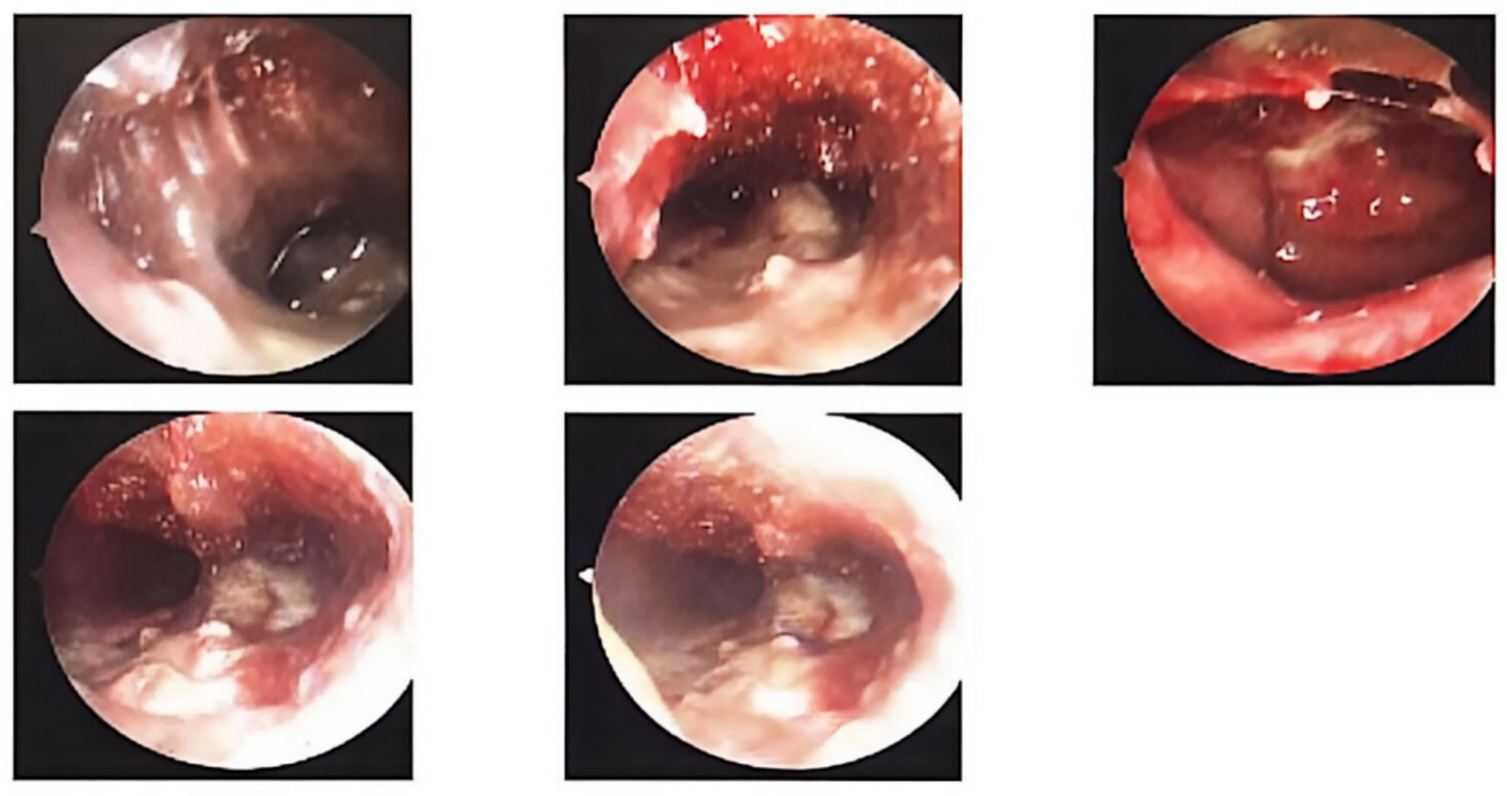
Nasal endoscopy on postoperative day.

On the 16th day of hospitalization, the child’s nasal congestion improved, CRP dropped to 20.22 mg/L, Hb was 84 g/L, and blood potassium was 2.41 mmol/L. Potassium chloride tablets (1 g q6h po) were given to replenish potassium. Every 3 to 4 days, nasal endoscopy was performed to explore and clean the surgical cavity, and no new infection foci or necrotic tissues were found. Nasal endoscopy on postoperative day 4 is presented in Figure 4.

On the 26th day of hospitalization, the patient’s sensation around the alae nasi to the upper lip partially recovered, and the sense of smell improved. The patient complained of headache and dizziness. The routine and biochemical tests of the cerebrospinal fluid obtained by lumbar puncture were negative, and no obvious abnormalities were found on cranial MRI, but the electroencephalogram remained abnormal. The re-examination of blood potassium was 3.38 mmol/L; potassium chloride tablets (1.5 g q6h po) were continued to strengthen potassium supplementation.

On the 29th day of hospitalization, facial and periorbital swelling completely subsided, the sense of smell was basically restored, and the sense of touch from the ala nasi to the upper lip further improved. Headache and dizziness disappeared, and no new positive neurological signs were found on physical examination. Cerebrospinal fluid bacterial culture, fungal culture, and mNGS were all negative. CRP was 11.34 mg/L, Hb was 83 g/L, and K^+^ was 3.29 mmol/L. Sputum culture was negative. Meropenem was discontinued. Potassium supplementation was adjusted to potassium chloride sustained-release tablets (2.5 g q8h po). Thereafter, nasal endoscopy was performed every 5–7 days to explore and clean the surgical cavity. No necrotic tissue, pseudomembrane regeneration, bleeding, or granulation hyperplasia was found, indicating that local infection was continuously and stably controlled.

On the 36th day of hospitalization, the child was conscious and in good spirits. The facial swelling had largely subsided, and the sense of smell had returned. The sensation from the ala nasi to the upper lip on the right side had fully recovered, while on the left side, it had improved compared to before. The re-examination showed CRP at 5.76 mg/L, Hb at 94 g/L, and blood potassium at 4.11 mmol/L. Second-generation sequencing of blood pathogenic microorganisms was negative. The course of liposomal amphotericin B had reached 4 weeks. Due to drug-related anemia (Hb 94 g/L), the pharmacist suggested switching from liposomal amphotericin B to oral isavuconazole capsules for sequential treatment (200 mg q8h po for the first 2 days; 200 mg qd po from the third day onwards), combined with local nasal drops of amphotericin B deoxycholate (2.5 mg/mL). Blood glucose fluctuated between 7 and 14.5 mmol/L. The hypoglycemic regimen was adjusted to a continuous subcutaneous insulin aspart infusion of 10–13 U for 24 h and subcutaneous injections of 6–4-7 U before each meal. Blood potassium was normal, and potassium supplementation was stopped.

On the 44th day of hospitalization, vital signs were stable, with no fever, headache, nasal congestion, or facial pain. The facial swelling has subsided; the sensation and olfactory function in the area from the ala nasi to the upper lip have both recovered; no positive neurological signs were found on physical examination. After a multidisciplinary assessment, the patient was discharged.

Outpatient follow-up: after discharge, the child continued to take oral isavuconazole capsules and amphotericin B deoxycholate for local nasal drops. Nasal endoscopy and surgical cavity cleaning were performed every 1 to 2 weeks, and no new foci of infection were found. Follow-up was conducted for 4 months, during which the electroencephalogram returned to normal, blood glucose was stable (5–10 mmol/L), liver and kidney functions and electrolytes were all normal, and the infection was well controlled.

## Discussion

Mucormycosis in children is rare but progresses rapidly and has a high fatality rate: 64% in neonates and 42%–56% in children (4). Etiologically, Rhizopus species are responsible for over 70% of mucormycosis cases worldwide (5, 6). The risk factors, predilection sites, and clinical manifestations of mucormycosis vary by age. The main underlying susceptibility factors are hematological malignancies (46%) and hematopoietic stem cell transplantation, others include malignant neoplasms, neutropenia, solid organ transplantation, diabetes mellitus, trauma or surgery, and preterm birth (7, 8). Diabetes is the primary risk factor in adults. Still, it is rare in children, type 1 diabetes accounts for only 4.8% of cases in children (7)^.^ The clinical types of pediatric mucormycosis vary depending on the underlying disease: disseminated and pulmonary types are more common in children with hematological malignancies and those undergoing transplantation; skin soft tissue and gastrointestinal types are more prevalent in children compared to adults, related to medical procedures; the nasal-orbital-brain type often follows diabetic ketoacidosis (9).

This case involved a 14-year-old patient with type 1 diabetes mellitus and diabetic ketoacidosis (DKA), who had high-risk factors for mucormycosis. The symptoms of early sinusitis progressed to nasal-orbital-brain mucormycosis. The patient initially exhibited only fever, nasal congestion, and epistaxis mimicking sinusitis. With disease progression, periorbital and facial swelling, anosmia, and facial hypoesthesia appeared, consistent with the course of rhino-orbital-cerebral mucormycosis (ROCM). The etiology was confirmed as Rhizopus. Although brain MRI showed no significant abnormalities, early intracranial involvement was suspected based on the presence of maxillary nerve sensory disturbance and electroencephalogram (EEG) abnormalities in the patient. This suggests that for adolescents with poorly controlled diabetes, symptoms related to this disease should be monitored vigilantly for those with this condition.

Due to the limited clinical data on pediatric mucormycosis, the treatment strategies mainly follow those for adults. The core lies in early diagnosis, reversing predisposing factors (such as controlling blood sugar and correcting acidosis), timely surgical debridement, and initiating effective antifungal therapy (10).

Actively managing underlying diseases and reversing predisposing factors are prerequisites for treating mucormycosis, including controlling blood sugar, correcting acidosis, increasing granulocyte levels, and, where possible, minimizing or discontinuing immunosuppressive drugs. After the child in this case was diagnosed with diabetic ketoacidosis, immediate intensive insulin therapy was initiated to promptly correct acidosis and hyperglycemia, creating favorable conditions for subsequent anti-infective treatment.

Early and thorough debridement surgery is the cornerstone of treatment for ROCM and, when combined with antifungal therapy, can significantly improve patient survival (11). Debridement removes necrotic tissue, reduces local fungal burden, disrupts the invasive microenvironment, and creates favorable conditions for antifungals. However, debridement alone cannot eliminate all infiltrated hyphae; the two modalities act synergistically: debridement reduces fungal biomass, while antifungal agents eradicate residual pathogens and prevent recurrence. After the child was diagnosed, an endoscopic nasal debridement procedure was performed immediately. Postoperatively, regular endoscopic nasal examinations and cavity clearance were conducted. The debridement interval was gradually extended from the initial 3–4 days to 5–7 days after discharge, and then to 7–14 days during follow-up. No new infection foci or necrotic tissue were found during the follow-up period. 2 months after the surgery, the mucosa on nasal endoscopy had essentially achieved epithelialization.

Systemic antifungal drug therapy is the key to controlling mucormycosis infections. Both the ECIL and ECMM guidelines recommend intravenous amphotericin B liposome (5–10 mg/kg/d) as the first-line drug for treating mucormycosis, especially for those with central nervous system involvement or renal insufficiency. In cases of intolerance or initial treatment failure, posaconazole or isavuconazole can be used for salvage treatment. For critically ill cases, amphotericin B liposome, combined with posaconazole or isavuconazole, can be considered, but due to limited clinical data, it is not routinely recommended (10, 11). After the condition stabilizes, oral preparations can be used for sequential treatment: Due to the lack of oral amphotericin B preparations, ECCM recommends that children aged 4–12 years take oral posaconazole suspension at 18 mg/kg/d divided into 3 doses; for those over 13 years old, posaconazole enteric-coated tablets are preferred, refer to adult doses, starting with 300 mg bid on the first day, followed by 300 mg qd, and blood drug concentration needs to be monitored during the treatment (target 1–2.5 μg/mL) (10). Alternatively, isavuconazole (prodrug, high bioavailability) can be used for patients >13 years and >40 kg (200 mg tid for 2 days, then 200 mg qd), without routine monitoring (12). For ROCM, isavuconazole offers theoretical advantage over posaconazole due to good blood–brain barrier penetration (13, 14). For patients with post-sinusitis fungal infection, local administration of amphotericin B (e.g., nasal instillation) can be used as an adjunct: dissolve 50 mg of amphotericin B deoxycholate in 15 mL of sterile water or 5% glucose, and administer at 0.8 mg/day (6 times/week for 1 month), then 0.5 mg/d (6 times/week for 1 month) (15). In this case, the sequential treatment of the child used isavuconazole capsules combined with local nasal instillation of amphotericin B deoxycholate.

In children, amphotericin B use is limited by nephrotoxicity, electrolyte imbalances, and hematologic adverse effects (16, 17). However, with appropriate monitoring and management, toxicity can be controlled without compromising therapeutic efficacy. In this case, the child’s serum creatinine and blood urea nitrogen remained within normal limits throughout treatment. Although there was a transient increase in urinary β2-microglobulin and urinary α1-microglobulin, they decreased spontaneously without discontinuation of liposomal amphotericin B, which was considered to be related to the underlying diabetes rather than drug-induced kidney injury. In addition, a severe hypokalemia (minimum 2.41 mmol/L) occurred during liposomal amphotericin B therapy, but it was restored after discontinuation of the drug and active potassium supplementation. During the treatment period, the child also experienced a mild decrease in hemoglobin, which was well tolerated after supportive care and did not affect the continuity of antifung therapy.

The optimal duration of treatment for mucormycosis remains undetermined; the total course should be at least 12 weeks and individualized (18)^.^ In this case, preemptive therapy with liposomal amphotericin B was initiated before microbiological confirmation, and prompt radical debridement was performed after diagnosis. Liposomal amphotericin B was maintained at an effective antifungal dose (5 mg/kg/d) throughout, along with supportive care. After the patient’s condition stabilized and following 4 weeks of induction therapy with liposomal amphotericin B, the regimen was promptly switched to oral isavuconazole capsules for sequential therapy combined with intranasal amphotericin B deoxycholate, in order to shorten the duration of amphotericin B exposure and reduce cumulative toxicity. The child has received oral isavuconazole sequential therapy for 4 months, with a planned total course of 6 months. During this period, regular monitoring of liver function, electrolytes, and other parameters showed good tolerance without significant adverse reactions.

## Conclusion

This case of successfully treating adolescent diabetic ketoacidosis complicated with rhino-orbital-cerebral mucormycosis demonstrates the comprehensive treatment strategy of early identification, active surgery, precise medication, and close monitoring under the framework of multidisciplinary collaboration. The key aspects include initiating antifungal treatment preemptively based on early symptoms resembling sinusitis; the multidisciplinary team promptly carrying out radical debridement; intensifying insulin therapy to control blood sugar and correct acidosis; continuous monitoring and intervention of dose-dependent adverse reactions (such as hypokalemia and anemia) caused by amphotericin B liposome; and sequentially switching to oral isavuconazole after stabilizing the condition, achieving safe and effective de-escalation treatment.

## Data Availability

The original contributions presented in the study are included in the article/supplementary material, further inquiries can be directed to the corresponding author.
